# The Co-Ordination of Local Health Services

**Published:** 1923-11

**Authors:** 


### THE CO-ORDINATION OF LOCAL HEALTH SERVICES.

### 1.—WANTED: A PLAN FOR THE PUBLIC HEALTH.

THE content of the words "Public Health" is so vast that it is not at all surprising that Ministers of Health, who come and go with such rapidity, do not have time even to understand what it all means—still less to produce a plan for dealing with the weighty issues involved. When we have a Minister of Health who can settle down to his real job, we may hear something of the machinery which was to be put into motion, after the setting up of the Ministry of Health in 1919, for co-ordinating the work of the local authorities engaged in different forms of health administration, and for getting rid of the overlapping about which we heard so much at that time.

### A Very Big Thing.

One of the many Ministers, Sir Alfred Mond, having attained greater freedom and less responsibility, did, indeed, inform a *Daily News* representative some little time ago that when he was at the Ministry he had in mind a thorough inquiry into the whole subject of the Public Health services, in which he found conflicting and overlapping organisations wasting energy, power, money and time. The whole of the medical services of this country, he said, are in a state of chaos and muddle. Any reorganisation, said Sir Alfred Mond, should take into account the poor law infirmaries, the voluntary hospitals, the school doctors, the private medical service, welfare workers, and National Health Insurance. If he had referred also to the sanitary services and the various special State-aided services of recent years, such as Tuberculosis and Veneral Disease, he would have had an added reason for saying that this reorganisation is a very big thing. Nor is there any reason for a courageous Minister to flinch from producing a scheme. It is essentially his job.

### The Aim.

If health administration is to be efficient and economical, the aim must be that "there should be one local authority for all public health purposes in every place so that no area should be without an authority or have more than one." These words are quoted from the recommendations of the Royal Sanitary Commission which was appointed in 1869 and reported in 1871. They are just as sound now as they were then. But the popular conception of what Public Health involves is immeasurably bigger now than it was fifty years ago, and it must be recognised that any such generalisation as to the need for one health authority in every area may have to be subject from the outset to some very wide delegation and ultimately to some new definition of areas.

### Examination Needed.

There is no need, in our view, for any extensive public enquiry before a scheme of co-ordination of public health services is produced. The facts are known. All that is necessary is that they shall be marshalled and examined in consultation with the different authorities concerned. If the authorities whose interests would be affected are strong enough in opposition to this or that proposal, it may well happen that the public interest will go to the wall. But no particular interest can survive as against the public interest if that interest is aroused, nor can it fail to bend sooner or later to the force of public opinion.

### The Lesson of the War.

We propose in this short series of articles to state the facts as to the distribution of public health services, and it will appear from the assembling of these facts that a plan is needed for bringing them under one general control. In the war on dirt and disease, the same unity of plan is needed as in the war carried on with guns and aeroplanes. The lesson which it took us a long time to learn in the war against Germany was that there must be unity of command before there could be a successful prosecution of the war. So far as central government is concerned we may regard the principle as embodied in the appointment of a Minister of Health in 1919. We hope that the time is not far distant when we may see the Minister concentrating on health problems and producing his plan for bringing the forces arrayed against dirt and disease under one co-ordinated scheme in each area.

### Some Central Adjustments.

It will be inevitable that some further adjustments of duties between the central departments should be made before the Minister of Health can completely review all health services with the object of taking effective action. The medical work connected with the care of school children, under the Board of Education, industrial hygiene at present within the province of the Home Office, and the medical services for merchant seamen, which are the subject of Board of Trade Regulations, are illustrations of matters which may well have to come, sooner or later, under the control of the Minister of Health. His department might, on the other hand, usefully shed some activities connected with local government and some at present associated with the Poor Law which are rather remote from the subject of Health.

### Authorities to be Consulted.

The central adjustments to which we have referred are, however, outside the scope of our present subject, which is that of the co-ordination of the local health services. The County and County Borough Councils, the Urban and Rural District Councils, the Boards of Guardians and the National Health Insurance Approved Societies and Insurance Committees all at present work under the general supervision of the Minister of Health. He also is in consultation with the medical profession and representatives ot the Voluntary Hospitals. These several authorities cover pretty completely the field of local health work ; it is they in the main who would have to be consulted and more or less persuaded of the reasonableness of the

Minister's proposals before he could hope to proceed far in Parliament with a measure in which their co-operation (or their acquiescence) would be inevitable. It is our purpose to bring under review the nature of the health functions of these different sets of authorities, to attempt some estimate of the public expenditure involved, whether from rates or taxes, and then to examine the question how far co-ordination is practicable.

### Growth of Public Health Activities.

In the last fifty years, that is, since the report of the Royal Sanitary Commission to which we have referred, we have had a quickening of the public conscience in regard to the health of the community. We have said that the content of public health is vast. At the time of the recommendations of the Royal Sanitary Commission, it embraced mainly questions of environment, that great group of what we may classify as Sanitary questions. The more modern development is concerned with the health and conduct of the individual or of groups of individuals, and these may be said to comprise the Medical questions. These two groups of questions, the Sanitary and the Medical, are so closely allied and have such intimate reactions on one another that it is difficult to conceive in a well-ordered scheme of authorities wholly independent of one another having the responsibility for their administration. In the growth of the measures for dealing with these sanitary and medical matters—both essential branches of the public health—it is not possible to see the lines of any uniform plan. The most striking health measure of recent years, the National Health Insurance Act, 1911, in fact created an entirely new set of authorities for providing for the health of the working population, and though it contained various provisions for co-operating with the existing local authorities, these have been to all intents and purposes a dead letter.

### The First Big Question.

Not only is there an absence of plan in the distribution of health functions between the County Councils and the Urban and Rural District Councils, but during the period of their greatest activity in health matters, there has been a development of Poor Law Medical services notwithstanding the determination of the Legislature not to add to the functions of the Poor Law Guardians. The overlapping of the Poor Law services, medical and social, with those of other authorities was the subject of a special report to the Government by a strong Committee in 1918 and their recommendations and a brief examination of the Poor Law health functions will be dealt with in the next article. They constitute the first big question in the way of the reform of our public health machinery.

### A Memorial Ward at Halifax.

Mr. Joseph Whitaker, of Halifax, has generously offered to give a new ward to house the X-ray department at Halifax Royal Infirmary, at a cost of £4,000, in memory of his late parents, Mr. and Mrs. Richard Whitaker, and of his son, Dr. Fred Whitaker, who died on war service at Alexandria in October, 1916. The new ward is designed to contain a special equipment for the treatment of cancer.

